# Multimodal ophthalmic imaging of staphylococcus aureus bacteremia associated with chorioretinitis, endocarditis, and multifocal brain abscesses

**DOI:** 10.1016/j.ajoc.2019.100577

**Published:** 2019-12-14

**Authors:** Chiara Veronese, Marco Pellegrini, Chiara Maiolo, Mariachiara Morara, Grayson W. Armstrong, Antonio P. Ciardella

**Affiliations:** a Ophthalmology Unit, S. Orsola-Malpighi Hospital, University of Bologna, Bologna, Italy; b University of Bologna, Bologna, Italy; c Department of Ophthalmology, Massachusetts Eye & Ear Infirmary, Harvard Medical School, Boston, MA, USA

**Keywords:** Bacterial endocarditis, Brain abscess, Chorioretinitis, Methicillin-resistant staphylococcus aureus, Staphylococcus aureus bacteriemia

## Abstract

**Purpose:**

*Staphylococcus aureus* bacteriemia (SAB) as critical condition for the life and occasionally involves the eyes. The aim of this report is to describe the ocular involvement with multimodal imaging.

**Observations:**

A patient admitted for evaluation of acute onset of confusion, disorientation, and generalized malaise and found to have methicillin-resistant staphylococcus aureus (MRSA)-associated endocarditis and multifocal brain abscesses was evaluated by the ophthalmology service. The patient's visual acuity was 20/20 OU without relative afferent pupillary defect and normal intraocular pressures. Bedside anterior segment examination was normal. Posterior segment examination revealed intraretinal hemorrhages and Roth spots in the posterior pole of the right eye, and two deep well-defined focal white chorioretinal infiltrates and a hemorrhagic pigment epithelium detachment in the temporal quadrant of the left eye. Multimodal imaging was utilized to document these findings and ensure adequate antibiotic therapy.

**Conclusion:**

SAB has the potential for poor visual outcomes as well as significant morbidity and mortality. Multimodal imaging of SAB-related chorioretinitis allows for accurate diagnosis as well as assessment of response to antimicrobial therapy.

## 1 Introduction

*Staphylococcus aureus* bacteremia (SAB) is a serious infection associated with significant morbidity and mortality. SAB can lead to metastatic infections in as many as 13%–39%, with the most common sites affected being bones, joints, kidneys, and the lung.^1^ Performing an exhaustive search for metastatic infections in cases of SAB is crucial, as widespread metastatic infections can determine the duration of antibiotic therapy required and may necessitate adjuvant therapy such as incisional abscess drainage or surgery.^2^ Occasionally, SAB can involve the eyes.^3^ Ocular involvement in SAB is a critical condition which may result in significant vision loss. Ness and Schneider reported on three patients with endogenous methicillin-resistant staphylococcus aureus (MRSA) endophthalmitis with poor visual prognoses,^4^ whereas a more recent case series of eight involved eyes of seven patients described better visual outcomes overall after successful treatment.^5^

We present the first description of multimodal imaging findings in a case of SAB-associated with chorioretinitis. We believe these results will aid ophthalmologists in accurate diagnosis and management of SAB-associated endophthalmitis.

## 2 Case report

**Fig. 1:**
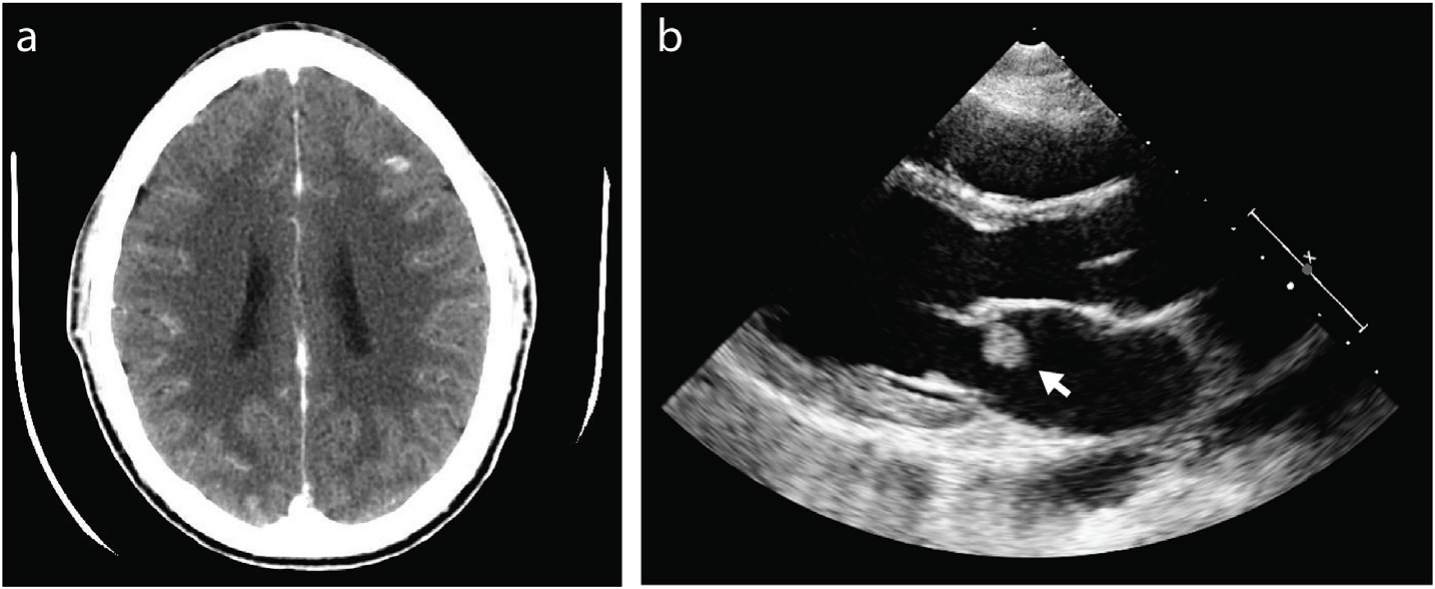
Computed tomography (CT) imaging of the brain showing multiple areas of abscesses (a) and echocardiography revealing mitral valve endocarditis (b, arrow).

A 28-year-old man was admitted to our hospital for evaluation of acute onset confusion, disorientation, and generalized malaise. Workup revealed MRSA bacteremia resulting in bacterial endocarditis and multifocal brain abscesses confirmed by computed tomography (Fig. 1). His medical history was unremarkable, and no underlying etiology of the bacteremia was identified. The patient denied a history of illicit drug use, recent dental work, or indwelling venous catheter.

The ophthalmology service was consulted for evaluation of the patient. At initial examination, the patient's visual acuity was 20/20 in both eyes, there was no relative afferent pupillary defect, and intraocular pressures were normal. The bedside anterior segment examination was normal without hypopyon formation in either eye. The vitreous was clear in both eyes. Posterior examination of the right eye revealed intraretinal hemorrhages and Roth spots of the posterior pole, while the left eye demonstrated two deep, well-defined, focal white chorioretinal infiltrates in the posterior pole as well as a hemorrhagic pigment epithelium detachment (PED) in the temporal quadrant. Given this constellation of findings in this clinical setting, a diagnosis of bilateral SAB associated with chorioretinitis was made.

Multimodal imaging of the retina was performed at initial evaluation, including fundus photo color (FP), multicolor images (MCI), fundus autofluorescence (FAF), fluorescein angiography (FA), indocyanine green angiography (ICGA), and spectral-domain optical coherence tomography (SD-OCT).

**Fig. 2:**
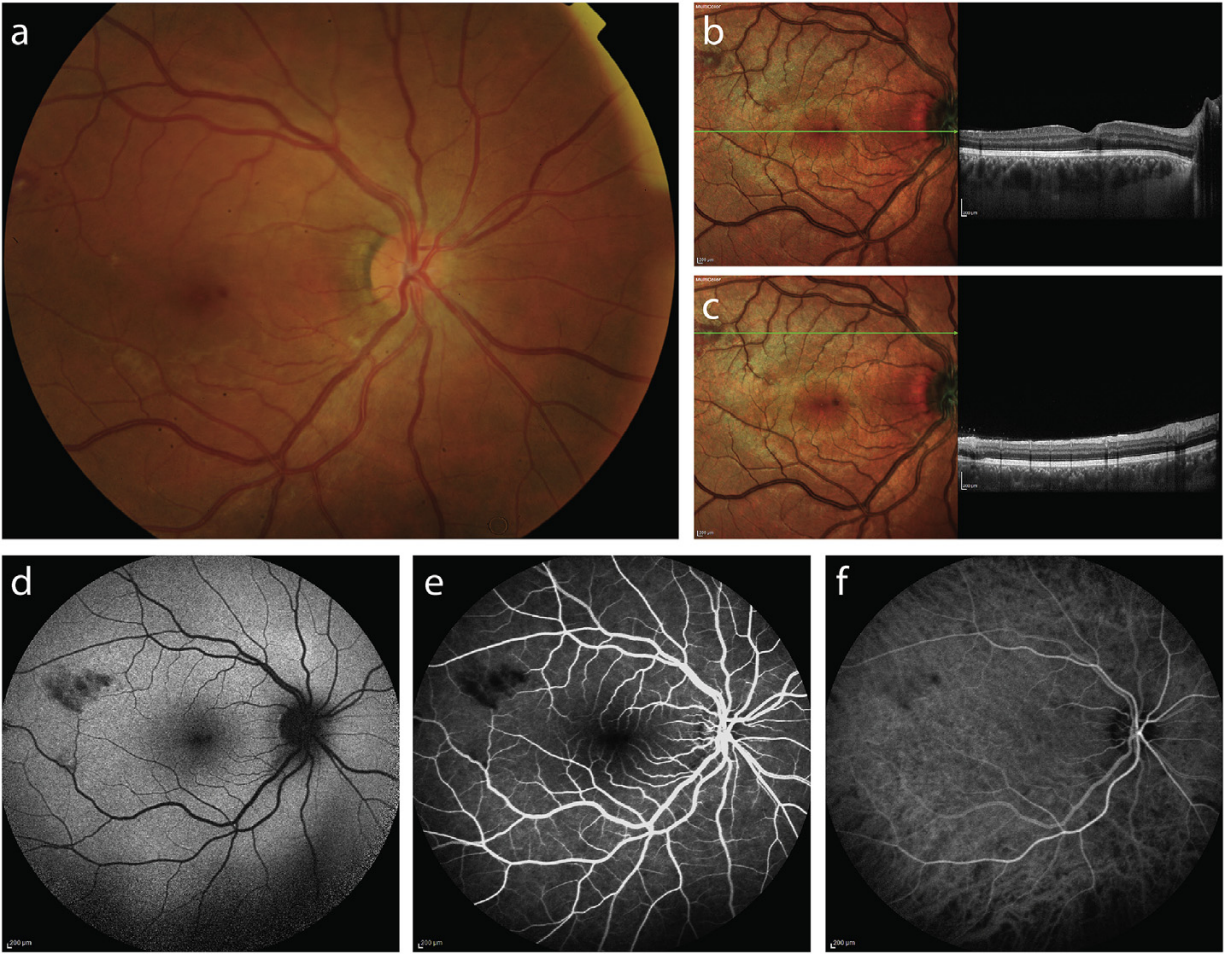
Multimodal imaging of the right eye of a patient with infectious chorioretinitis secondary to staphylococcus aureus bacteriemia. Fundus color photography (a) and multicolor images (b,c) reveal hemorrhages and Roth spots of the posterior pole, while spectral-domain optical coherence tomography (b,c) highlights hyperreflective areas in the superficial retinal layer. Fundus autofluorescence (d) identified hypofluorescent areas corresponding to intraretinal hemorrhages. Fluorescein angiography (e) revealed normal retinal vasculature with hypofluorescent lesions corresponding to hemorrhages. Indocyanine green angiography (f) demonstrates blockage associated with intraretinal hemorrhages.

**Fig. 3:**
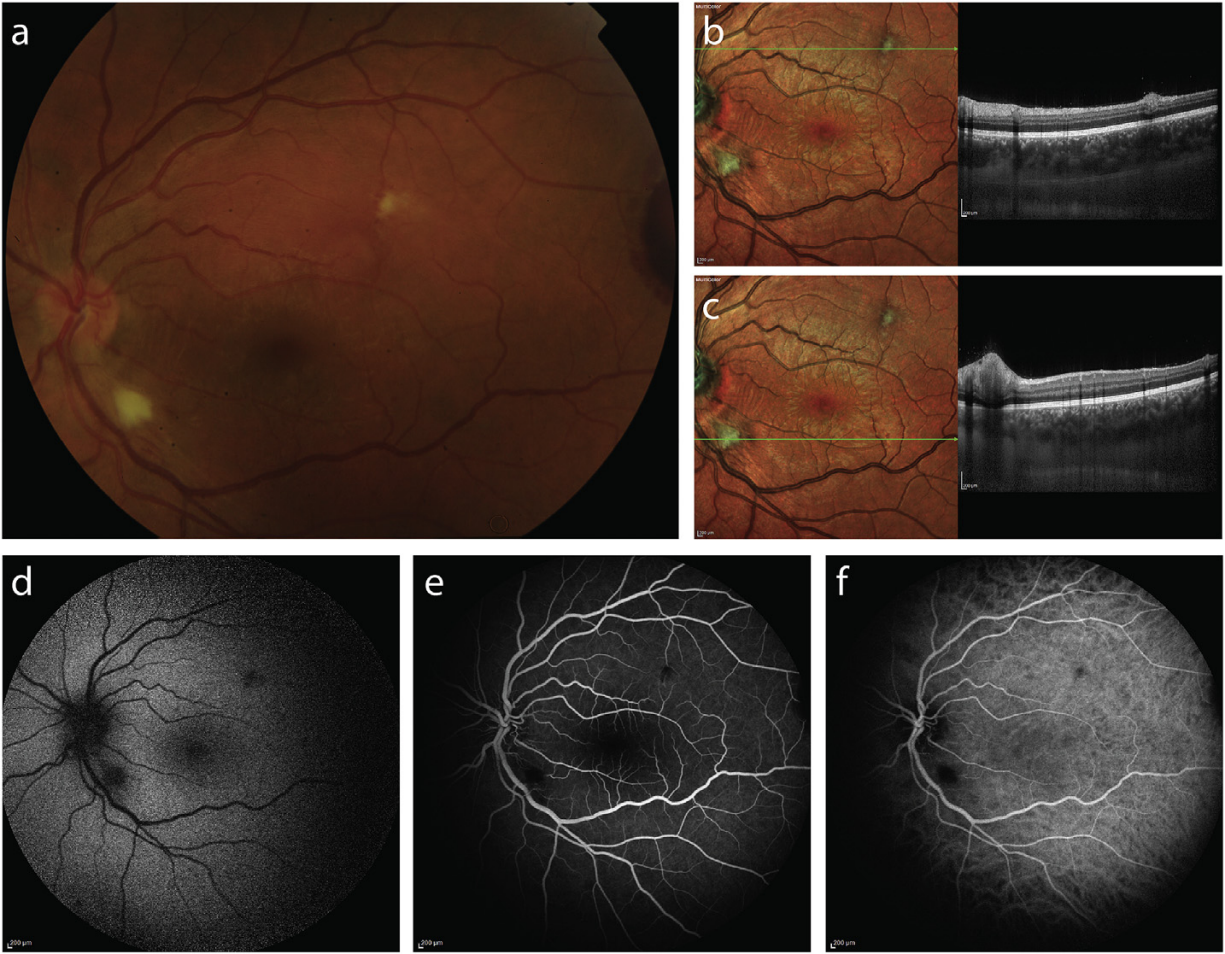
Multimodal imaging of the left eye of a patient with infectious chorioretinitis secondary to staphylococcus aureus bacteriemia. Fundus color photography (a) and multicolor images (b,c) showed two deep, well-defined, focal yellow infiltrates corresponding to homogenous hyperreflective lesions on spectral-domain optical coherence tomography (b,c). Fundus autofluorescence (d) identified two round hypofluorescent lesions, which appeared hypofluorescent on fluorescein angiography (e) and with blocking on indocyanine green angiography(f), corresponding to chorioretinal infiltrates.

The retinal hemorrhages and Roth spots of the right eye were more prominent on MCI than on FP (Fig. 2 a-c); while the two deep, well-defined, focal white chorioretinal infiltrates and the hemorrhagic PED in the temporal quadrant was well visualized on both FP and MCI (Fig. 3 a-c). FAF identified hypofluorescent areas corresponding to intraretinal hemorrhages in the right eye (Fig. 2 d) and two round hypofluorescent lesions corresponding to the chorioretinal infiltrates in the left eye (Fig. 3 d). FA revealed normal retinal vasculature with hypofluorescent lesions corresponding to hemorrhages (Fig. 2 e) and chorioretinal infiltrates (Fig. 3 e). ICGA demonstrated areas of blockage in areas of intraretinal hemorrhages (Fig. 2 f) and infiltrates (Fig. 3 f). SD-OCT of the right eye revealed hyperreflective areas in the superficial retinal layer (Fig. 2 b,c), and two round homogenous hyperreflective lesions corresponding to areas of SAB chorioretinitis in the left eye (Fig. 3 b,c).

**Fig. 4:**
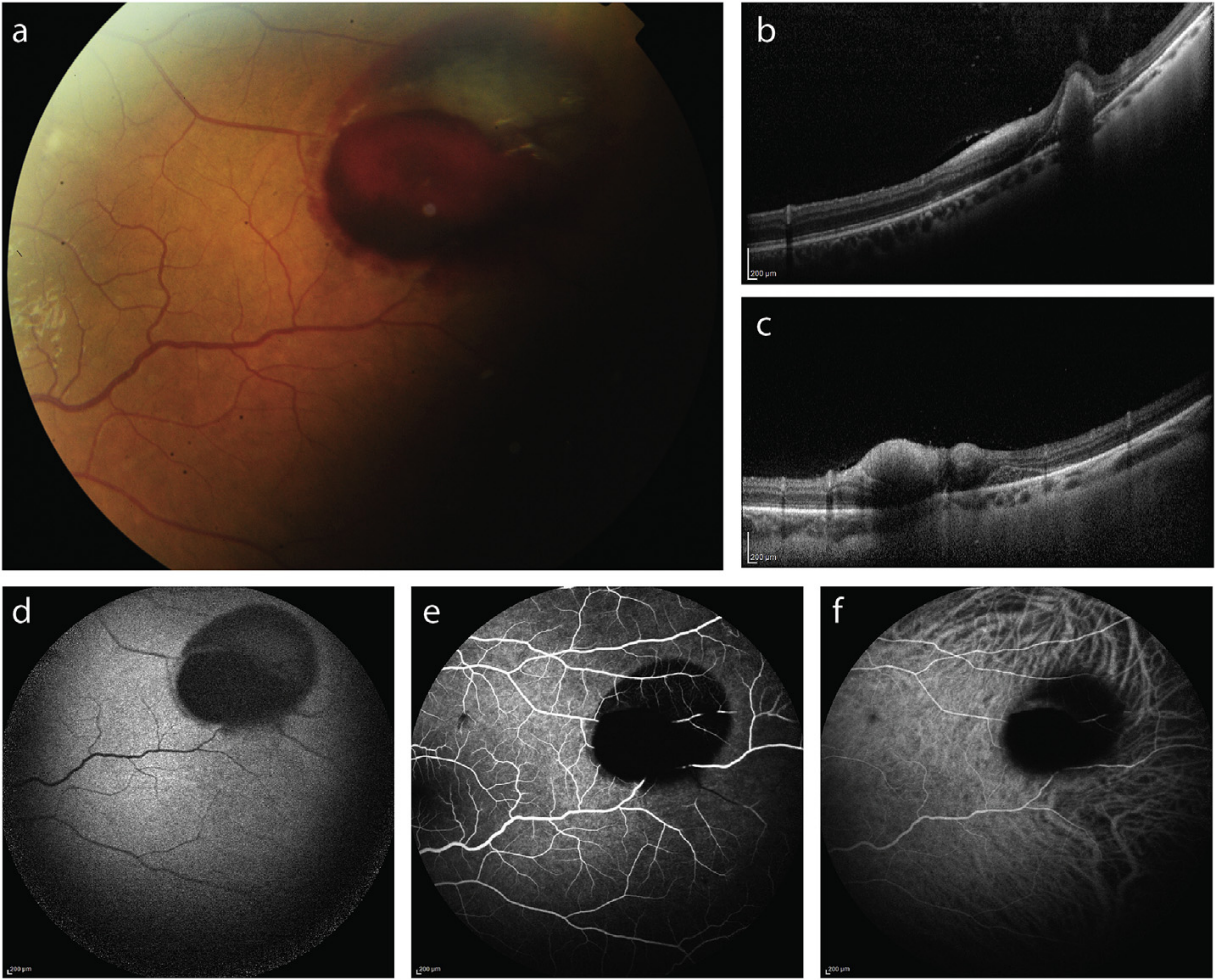
Multimodal imaging of the temporal quadrant of the left eye of a patient with infectious chorioretinitis secondary to staphylococcus aureus bacteriemia. Fundus color photography (a) revealed an hemorrhagic pigment epithelium detachment and a subhyaloid hemorrhage corresponding to hyperreflective subretinal and preretinal lesions on spectral-domain optical coherence tomography, respectively (b,c). Fundus autofluorescence (d), fluorescein angiography (e) and indocyanine green angiography (f) identified hypofluorescent lesions.

In the temporal quadrant of the left eye, multimodal imaging revealed a hemorrhagic PED and subhyaloid hemorrhage on FP (Fig. 4 a). SD-OCT confirmed the presence of a hemorrhagic PED (Fig. 4 b) as subhyaloid hemorrhage (Fig. 4 b,c). FA and ICGA revealed blockage of retinal arteries associated with subhyaloid hemorrhage as well as blockage of choroidal circulation caused by the hemorrhagic PED (Fig. 4 d,e). FAF revealed hypofluorescence in areas of pre- and sub-retinal hemorrhage (Fig. 4 f).

Systemic therapy with intravenous oxacillin (12 gr daily) and oral levofloxacin (1 gr daily) was administered. One week later the patient underwent surgical intervention to replace his infected cardiac valve.

## 3 Discussion

Ocular involvement in SAB is vision-threatening condition warranting prompt diagnosis and management. In a recent study, Jung et al. reported that ocular involvement was not uncommon among patients with SAB and was associated with increased 30-day mortality and 12-week mortality rates.^6^ Therefore, routine ophthalmic examinations should be considered in patients with SAB, especially those with infectious endocarditis or metastatic infections. Despite the potential for devastating vision loss, our patient experienced good visual outcomes despite bilateral chorioretinal involvement. To our knowledge, this is the first report of chorioretinitis analyzed by multimodal ophthalmic imaging techniques. Non-invasive imaging studies such as those presented here can assist in diagnosis and management of patients with SAB-associated ophthalmic infections and can help clinicians determine response to systemic antibiotic therapy and the potential need for more invasive treatment such as intravitreal antiobiotic administration or pars plana vitrectomy.

## 4 Conclusions

SAB has the potential for poor visual outcomes as well as significant morbidity and mortality. Multimodal imaging of SAB-related chorioretinitis allows for accurate diagnosis as well as assessment of response to antimicrobial therapy. Accordingly, we recommend routine ophthalmic examinations in patients with SAB in order to ensure prompt aggressive treatment with systemic antibiotics to prevent potential vision loss.

## Patient consent

The patient consented to publication of the case in writing.

## Funding

No funding or grant support.

## Authorship

All authors attest that they meet the current ICMJE criteria for Authorship.

## Declaration of competing interest

The following authors have no financial disclosures: CV, MP, CM, MM, GWA, APC.
